# A Novel Prognostic Model for Identifying the Risk of Hepatocellular Carcinoma Based on Angiogenesis Factors

**DOI:** 10.3389/fgene.2022.857215

**Published:** 2022-03-18

**Authors:** Yuan Gao, Jia Liu, Dexi Zhao, Guanghao Diao

**Affiliations:** Department of Hepatobiliary Surgery, the Fifth Medical Center of Chinese PLA General Hospital, Beijing, China

**Keywords:** hepatocellular carcinoma, angiogenic factor, nomogram, risk score, prognosis

## Abstract

Hepatocellular carcinoma (HCC) is the most common primary liver cancer with poor prognosis. An optimized stratification of HCC patients to discriminate clinical benefit regarding different degrees of malignancy is urgently needed because of no effective and reliable prognostic biomarkers currently. HCC is typically characterized by rich vascular. The dysregulated vascular endothelial growth factor was proved a pivotal regulator of the development of HCC. Therefore, we investigated the capability of angiogenic factors (AFs) in stratifying patients and constructed a prognostic risk model. A total of 6 prognostic correlated AFs (*GRM8*, *SPC25*, *FSD1L*, *SLC386A*, *FAM72A* and *SLC39A10*) were screened via LASSO Cox regression, which provided the basis for developing a novel prognostic risk model. Based on the risk model, HCC patients were subdivided into high-risk and low-risk groups. Kaplan-Meier curve indicated that patients in the high-risk group have a lower survival rate compared with those in the low-risk group. The prognostic model showed good predictive efficacy, with AUCs reaching 0.802 at 1 year, 0.694 at 2 years, and 0.672 at 3 years. Univariate and multivariate cox regression analysis demonstrated that the risk score had significant prognostic value and was an independent prognostic factor for HCC. Moreover, this model also showed a good diagnostic positive rate in the ICGC-LIRI-JP and GSE144269. Finally, we demonstrated the efficacy of the AF-risk model in HCC patients following sorafenib adjuvant chemotherapy. And revealed the underlying molecular features involving tumor stemness, immune regulation, and genomic alterations associated with the risk score. Based on a large population, we established a novel prognostic model based on 6 AFs to help identify HCC patients with a greater risk of death. The model may provide a reference for better clinical management of HCC patients in the era of cancer precision medicine.

## Introduction

Hepatocellular carcinoma (HCC), as the most common primary liver cancer, is amalignant tumor with poor prognosis (Craig et al., 2020). HCC is currently the fifth most common cancer and the second leading cause of cancer-related death worldwide (Degasperi and Colombo, 2016). HCC accounts for more than 80% of primary liver cancers worldwide (Global Burden of Disease Cancer et al., 2017). In the past few decades, considerable progress has been made in prevention, surveillance, early detection, diagnosis, and treatment of HCC. However, the incidence rate and cancer-specific mortality rate of HCC continue to increase in many countries (Yang et al., 2019). Indeed, the current 5-years survival rate for HCC is no more than 20% (El-Serag, 2011; Li et al., 2020) and early diagnosis is important for the treatment of HCC patients (Li et al., 2020). Novel prognostic biomarkers are urgently needed because there have been no effective and reliable prognostic biomarkers for HCC patients. Therefore, it is critical to develop a multi-dimensional model to identify patients at high risk and aim to achieve personalized medicine in HCC patients.

Tumor’s access to the blood system is mainly accomplished by sprouting angiogenesis (Hillen and Griffioen, 2007). Angiogenesis is an essential hallmark and is induced surprisingly early in cancer development (Hanahan and Weinberg, 2011). The tumor microenvironment utilizes numerous signaling factors that regulate the angiogenic response (Weis and Cheresh, 2011). Inhibition of angiogenesis has become an established treatment strategy for many solid tumors (Li et al., 2019). Angiogenic factors (AFs) include pro- and anti-AFs to keep angiogenesis in balance, while breaking this equilibrium can turn on the switch of angiogenesis, which act as a prerequisite for growth and metastasis of tumor (Bergers and Benjamin, 2003; Baeriswyl and Christofori, 2009).

For example, WNT2 has been confirmed to correlate with prognosis and considered to be an angiogenic growth factor that promotes liver regeneration (Klein et al., 2009; Ding et al., 2010). Expressions of the pro-angiogenic cytokines were also founded to be associated with outcomes of patients with advanced hepatocellular carcinoma (Miyahara et al., 2013). Therefore, we attempted to establish a risk model using AF genes to evaluate the prognosis of HCC and further help develop new treatment strategies.

In this study, we constructed and verified an effective prognostic risk model based on the expression of informative AFs. The investigation of the risk score deepened further understanding of the divergence of molecular features underlying different risk groups. This model was also proved to be suitable for patients following sorafenib adjuvant chemotherapy and we created the predictive nomogram. As a whole, this prognostic model might help guide the prognostic status of patients with HCC.

## Materials and Methods

### Data Acquisition and Preprocessing of HCC Samples

The raw counts of RNA-Seq data and corresponding clinical information of TCGA-LIHC patients were collected as the training cohorts. Data were downloaded from UCSC Xena (http://xena.ucsc.edu/). We also obtained two independent validation cohorts of HCC patients from the ICGC portal (ICGC-LIRI-JP, https://dcc.icgc.org/projects/LIRI-JP) and the GEO (GSE144269, https://www.ncbi.nlm.nih.gov/geo/), with transcriptomic and clinical data available.

### AFs Genes Collection

We first collected genes with annotations related to “angiogenesis” in NCBI. Then the signature gene sets of “angiogenesis” from the MSigDB database were also obtained (http://www.gsea-msigdb.org/gsea/msigdb/collections.jsp). The final AFs gene set studied here was the combination of genes from the two resources (Supplementary Table S1).

### Differential Expression Analysis of the AFs Genes

In order to obtain the differential expressed genes between tumor and normal samples, Log-transformed fold change (FC) and FDR of each gene was analyzed by DESeq2 (version 1.34.0) package (Love et al., 2014). The two “recurrent solid tumor” samples were removed from analysis. Genes with FDR < 0.05 and | log2 FC|>1 were defined as differentially expressed genes.

### Survival Analysis

Univariate Cox regression was performed for each AF genes to obtain the prognostic genes with *p*-value < 0.01 and HR > 1 or HR < 0.5. Kaplan-Meier analysis was also performed to screen the prognostic candidate AF genes using R package survival (version 3.2-13) (Borgan and Therneau, 2001). Multivariate Cox regression was used to assess the performance of risk score under the effects of other clinical factors.

### Construction of the AFs-Derived Prognostic Risk Model

The prognostic candidate AF genes were screened by univariate cox regression and log-rank test. Tumor samples of the TCGA-LIHC were used as the training cohort to establish the LASSO model. A lasso penalty was used to find the best gene model utilizing an R package glmnet. The risk score for each sample can be calculated with the final LASSO model coefficient as follows:

$$Risk\;Score=\;\overset{n}{\underset{i=1}{\sum }}\exp ression\;of\;gene\;i*lasso\;coefficient\;of\;gene\;i$$

The median risk score was used as cutoff for high-risk group (with higher risk score) and low-risk group (with lower risk score).

### Molecular Features of HCC Samples

CIBERSORT algorithm was used to evaluate the infiltration of 22 immune cell types (Newman et al., 2015). The immune score and stromal score were tested by the R package ESTIMATE (Yoshihara et al., 2013). TIDE was used to predict the potential of patients to response for immunotherapy (Jiang et al., 2018). We also collected tumor stemness score, TMB and HRD score of TCGA tumors from previous studies (Chen et al., 2021). mDNAsi, EREG-mDNAsi, DMPsi and ENHsi data of TCGA-LIHC tumor samples were collected from exist studies (Malta et al., 2018).

### SNV and CNV Mutation Analysis

Mutation comment file (MAF) of TCGA-LIHC cohort was downloaded from the GDC client. Differential analysis and visualization of somatic mutations were performed using maftools package. The Fisher’s exact test was used on all genes between two groups to detect differentially mutated genes. Segment file of the TCGA-LIHC cohort was downloaded from FIREHOSE and analyzed using the GISTIC 2.0 pipeline (Mermel et al., 2011).

### Nomogram Construction Based on AFs-Derived Prognosis Risk Model

AFs-derived risk scores, TNM stages, clinical stage, gender, age and grade were used as independent prognostic factors through univariate cox regression and AFs-derived prognosis risk model. Nomogram was finished based on the results of multivariate cox regression analysis. The calibration curves of the nomogram were constructed to test consistency between 1-, 3- and 5-years survival probability prediction and actual observation. The performance of the nomogram was evaluated using the concordance index (C-index) and time-dependent receiver operating characteristic (ROC) curves. Nomograms analysis and visualization were performed using R packages rms (version 6.2-0) (JrHarrell, 2021) and survival (version 3.2-13) (Borgan and Therneau, 2001) with default parameters.

## Results

### The Construction of AFs-Derived Prognostic Risk Model

**FIGURE 1:**
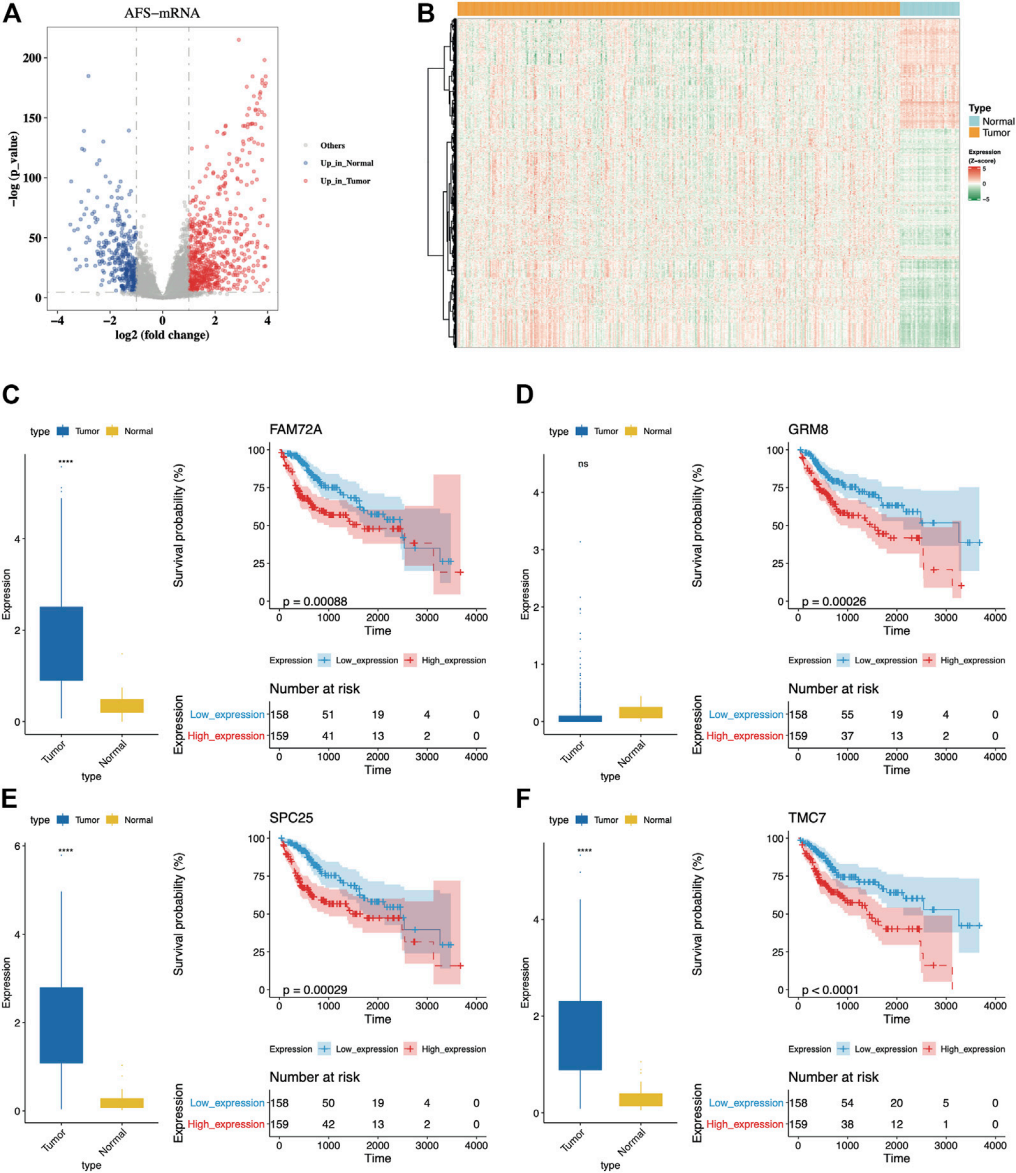
Dysregulated AFs could distinguish OS of patients. (**A)** The volcano plot of differential expressed AFs genes between tumor and normal samples from the TCGA-LIHC cohort. The red dots indicated upregulated genes while blue dots indicated downregulated genes in tumors. **(B)** The distribution of expression levels of the differential expressed AFs genes. **(C-F)** Comparison of expression levels between tumor and normal samples and K-M curves for the four genes with top significance. The prognostic group was separated using the median expression of each gene as the cutoff.

First, the large population of liver hepatocellular carcinoma (LIHC) patients from the TCGA database was used as the training cohort. We downloaded the transcriptomic and clinical data for 371 tumor samples and 50 adjacent normal samples. A total of 8,250 differentially expressed genes were found between tumor and normal samples (FDR <0.01, |log2FC| > 1, DEseq2). We then systematically collected AF genes from NCBI and MSigDB (see Methods) and noticed 1,038 AFs were differentially expressed (hereafter termed DE-AFs, Figures 1A,B). Among them, 361 DE-AFs were downregulated and 677 DE-AFs were upregulated in tumors.

Then we investigated the prognostic ability and regression characteristics of DE-AFs using univariate Cox regression analysis. A total of 36 genes with significant differences were discovered in univariate regression model (*p*-value < 0.01, hazard ratio (HR) > 1 or HR < 0.5, Supplementary Table S2). Furthermore, the Kaplan-Meier curve with log-rank test was also used to filter important DE-AFs and finally screened 17 genes with confirmed prognostic efficacy (log-rank *p*-value < 0.05, Supplementary Figures S1 and S2), including *EGF*, *GRM8*, *TRPM6*, *SLC38A6*, *BLM*, *BARD1*, *CLSPN*, *PRIM2*, *MSH2*, *FAM72A*, *SPC25*, *IGF2BP3*, *CENPP*, *GTF2IRD1*, *TMC7*, *FSD1L*, and *SLC39A10*. The distribution of expression levels and Kaplan-Meier curves of the four genes with top significance were displayed (Figures 1C–F). It was obvious that higher expression of them indicated poor prognosis of patients.

**FIGURE 2:**
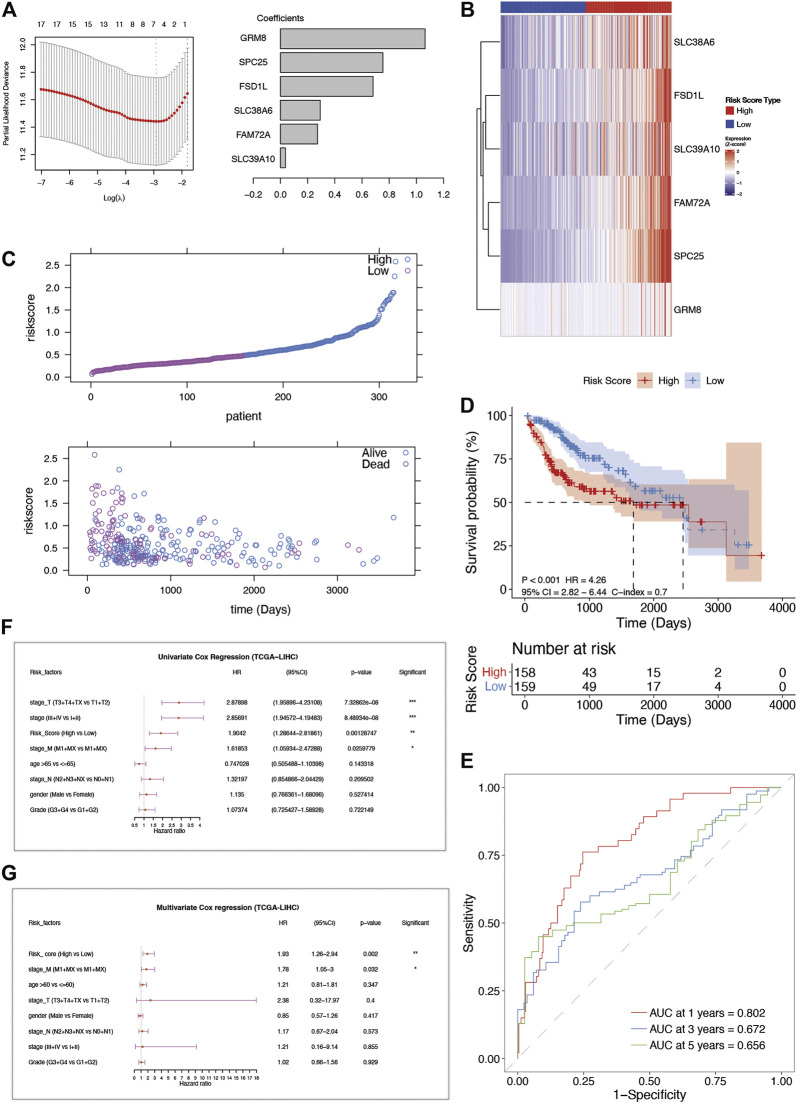
The construction and verification of AFs-derived prognostic model in TCGA-LIHC cohort. **(A)** LASSO coefficient profiles of the selected AFs genes. **(B)** The heatmap of z-score transformed gene expression for the 6 selected AFs genes. **(C)** The risk score distribution and survival status distribution of the AFs-derived prognostic model in TCGA -LIHC cohort. **(D)** Kaplan–Meier OS curve with log-rank test for risk score. **(E)** ROC curves of the risk score in predicting 1-, 3-, and 5-years OS. **(F)** Univariate and **(G)** multivariate Cox regression analysis of risk score and clinical factors.

Although these genes showed certain predictive efficacy by means of the intersection of univariate Cox regression analysis and log-rank test, we preferred combining the informative genes to obtain a more optimized prognostic model. The LASSO regression analysis was subsequently performed to remove redundant factors and also filtered the factors with less contribution. The 6 AFs with the most predictive value were selected (Supplementary Figures S3 and S4), including *GRM8*, *SPC25*, *FSD1L*, *SLC38A6*, *FAM72A,* and *SLC39A10*. In addition, several publications also supported these genes in cancer with experimental evidence (Supplementary Table S3), such as siRNA approach. Then a prognostic risk scoring model of AFs with the coefficients from LASSO regression analysis was constructed (Figure 2A). Their expression was accordantly upregulated to define the high-risk group of HCC (Figure 2B).

Based on the 6-AFs gene prognostic model, patients of TCGA-LIHC were stratified into high-risk group (*n* = 158) and low-risk group (*n* = 159) according to the median cut-off value of risk score. The overall survival (OS) time of patients in the high-risk group was remarkably decreased (Figure 2C). The Kaplan-Meier and C-index analysis showed the capacity of the AFs prognostic model (log-rank test *p*-value < 0.001, C-index = 0.7, Figure 2D). The predictive performance of the prognostic risk model was further evaluated by time-dependent ROC curves, and the area under the ROC curve (AUC) reached 0.802 at 1-year, 0.694 at 2-years, and 0.672 at 3-years (Figure 2E).

After performing the univariate Cox regression analysis (Figure 2F), we explored the relationship between clinical characteristic factors and AFs risk score. Age, gender, stage, grade, and the risk score of the prognostic model were included in the multivariate Cox regression model. The risk score was found to be an independent predictor for OS, with HR = 1.93, 95% CI: 1.26–2.94, *p*-value = 0.002 (Figure 2G, Table 1). Taken together, the AFs gene prognostic model was confirmed as a credible and independent predictor of OS in HCC.

**TABLE 1:** Univariate and Multivariate Cox regression analysis of AFs-derived risk score and clinical factors in TCGA-LIHC cohort.

Factors	Univariate cox regression					Multivariate cox regression		
	Beta	HR	95%_CI	*p*-value	C-index	HR	95%_CI	*p*-value
Age	0.13	1.14	(0.77-1.68)	0.53	0.50	1.21	0.81-1.81	0.35
Gender	−0.29	0.75	(0.51-1.10)	0.14	0.52	0.85	0.57-1.26	0.42
Grade	0.07	1.07	(0.73-1.59)	0.72	0.52	1.02	0.66-1.56	0.93
Risk score	0.64	1.90	(1.29-2.82)	0.00	0.70	1.93	1.26-2.94	0.00
Stage	1.05	2.86	(1.95-4.19)	8.49E-08	0.62	1.21	0.16-9.14	0.86
Stage_M	0.48	1.62	(1.06-2.47)	0.03	0.53	1.78	1.05-3	0.03
Stage_N	0.28	1.32	(0.85-2.04)	0.21	0.49	1.17	0.67-2.04	0.57
Stage_T	1.06	2.88	(1.96-4.23)	7.33E-08	0.62	2.38	0.32-17.97	0.40

abrAbbreviationHR, hazard ratio; CI, confidence interval.

### Validation of AFs-Derived Prognostic Risk Model in Independent Datasets

**FIGURE 3:**
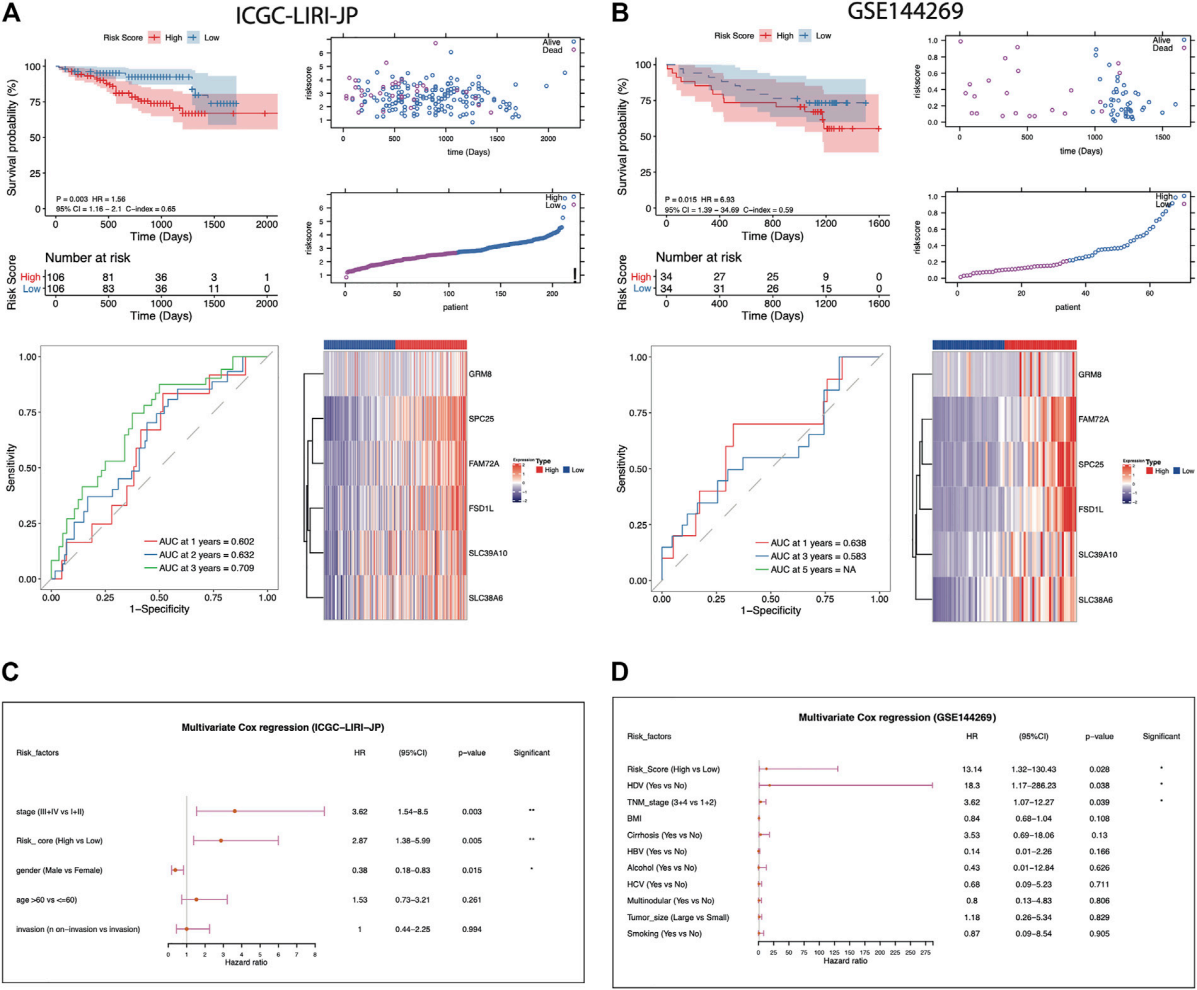
Evaluation of the AFs-derived prognostic model in ICGC-LIRI-JP and GSE144269 cohorts. **(A-B)** Visualization of the following analysis in ICGC-LIRI-JP and GSE144269 HCC cohorts, respectively. Kaplan–Meier curve with log-rank test for risk model. The risk score distribution and survival status distribution of the AFs-derived prognostic model. ROC curves of risk score in predicting 1-, 3-, and 5-years OS. The heatmap of z-score transformed gene expression for the 6 selected AFs genes. **(C-D)** Multivariate Cox regression analysis of risk score in the two independent validation cohorts.

To assess the robustness and generalizability of the AFs-derived prognostic risk model, the validation data sets from ICGC-LIRI-JP (*N* = 212) and GSE144269 (*N* = 68) were collected. Patients were all separated into high- or low-risk groups according to the risk score. The high-risk group of the ICGC-LIRI-JP validation cohort also showed significantly lower survival rate than the low-risk group (log-rank *p*-value = 0.03). The predictive capacity was proved as AUC reaching 0.602 at 1-year, 0.632 at 2-years, and 0.709 at 3-years in ICGC-LIRI-JP cohort (Figure 3A). Consistently, another validation set from GSE144269 (N = 68) also supported the poor prognosis of the high-risk group (log-rank *p*-value = 0.015, Figure 3B). Likewise, in the ICGC-LIRI-JP and GSE144269 cohorts, the risk score still proved to be an independent predictor for OS after correction for other confounding clinical factors (multivariate Cox regression analysis, ICGC-LIRI-JP cohort: HR = 2.87, 95%CI = 1.38-5.99, *p* = 0.005; GSE144269 cohort: HR = 13.14, 95%CI = 1.32-130.43, *p* = 0.028; Figures 3C,D).

### Comparison of Molecular Features Between Different Risk Groups

**FIGURE 4:**
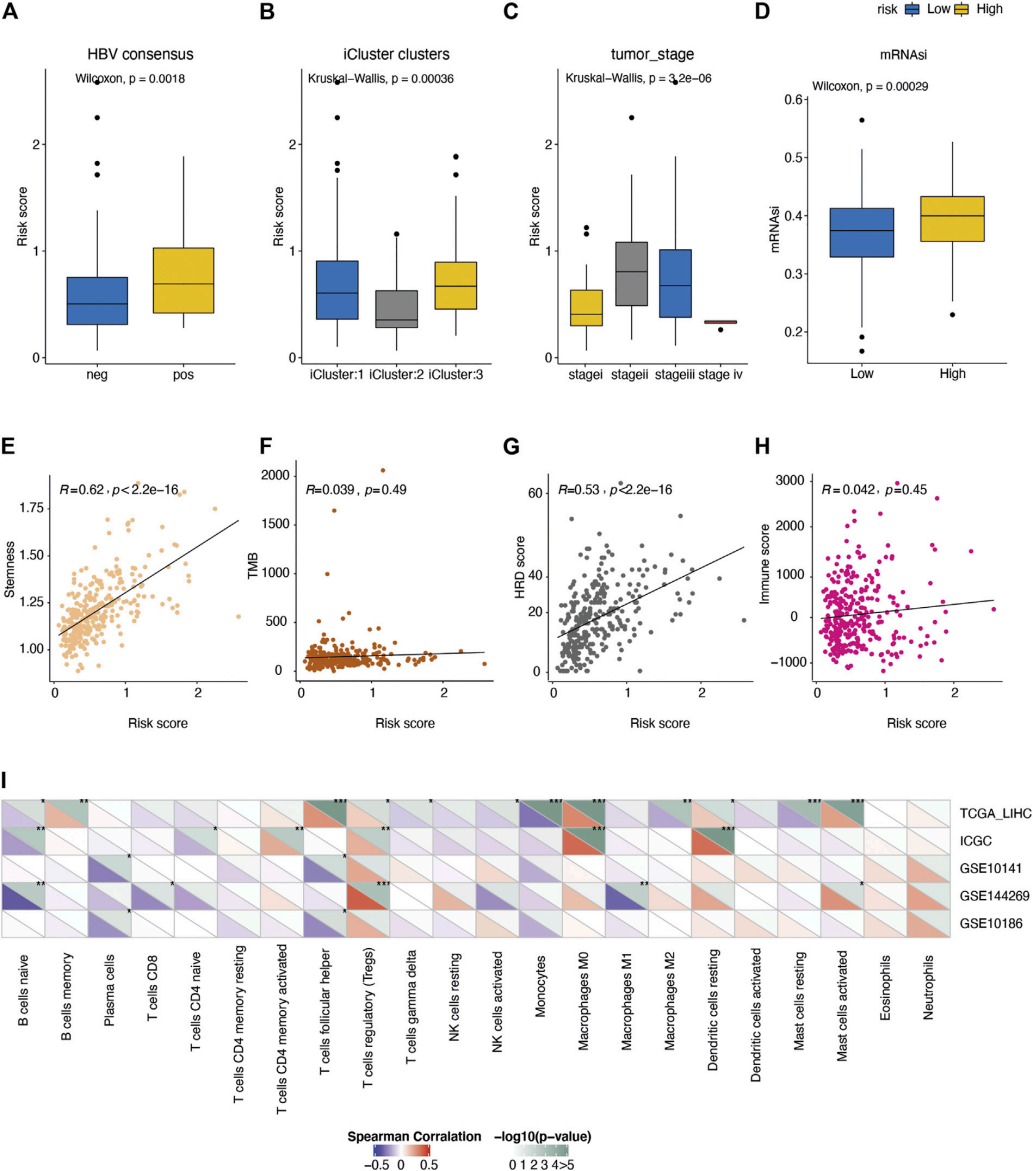
Comparison of the molecular features between different risk groups. Distribution of risk score under the status of **(A)** HBV infection, **(B)** TCGA molecular typing, **(C)** tumor stage. **(D)** Comparison of the tumor stemness score (mRNAsi) between high/low-risk groups. **(E-H)** Spearman correlation of the risk score with **I** tumor stemness score, **(F)** mutation burden, **(G)** HRD score, and **(H)** immune score. **(I)** Spearman correlation of risk score with fractions of 22 immune cell types in five liver cancer cohorts.

In order to explore the underlying molecular mechanisms of this AFs-derived prognostic risk model, we assessed the associations between the risk score and the typical clinical characteristics including age, gender, TCGA molecular typing (iclust1, iclust2, iclust3), tumor stage, virus infection status, *etc*. (Cancer Genome Atlas Resea, 2017). Chronic infection of Hepatitis B virus (HBV) has been commonly considered as a major risk factor in the initiation and development of HCC (Chan et al., 2016). We observed the AFs risk score was positively associated with the risk of HBV infection (Wilcoxon rank-sum test *p*-value = 0.0018, Figure 4A), which further confirmed its predictive value of severe disease status. Besides, the risk score varied in different TCGA molecular typing groups (Kruska-Wallis test *p*-value = 0.00036, Figure 4B). Higher risk score indicated a higher tumor stage, and the exception of stage IV was probably due to the limited samples size (Kruska-Wallis test *p*-value = 3.2e-6, Figure 4C). Previous analysis proved that cancer stem cells promoted angiogenesis by secreting factors such as vascular endothelial growth factor (VEGF) and stromal cell-derived factor 1 (SDF1). Therefore, we obtained two measurements depicting the tumor stemness (Malta et al., 2018; Chen et al., 2021), and verified a positive correlation between the tumor stemness and AFs risk score in TCGA-LIHC cohorts (Figures 4D,E).

Next, we investigated the associations between the AFs risk score and the immune response in tumors. The mutation burden (TMB) was not differed between high- and low-risk groups (Figure 4F), while the homologous recombination deficiency (HRD) score was positively correlated with the AFs risk score (Spearman R = 0.53, *p*-value < 2.2e-16, Figure 4G). This was in line with the moderate potential to induce adaptive immunity in high-risk group (Figure 4H). As for individual immune cell types, we calculated the relative fraction of 22 immune cell types in five cohorts (TCGA-LIHC, ICGC-LIRI-JP, GSE10141, GSE144269 and GSE10186) by the CIBERSORT algorithm. And found that the fraction of immunosuppressive regulatory T cells (Tregs) cells, M0 macrophages, and resting dendritic cells showed positive correlations with the risk score, while several adaptive immune cells showed the trends of negative correlations (Figure 4I).

**FIGURE 5:**
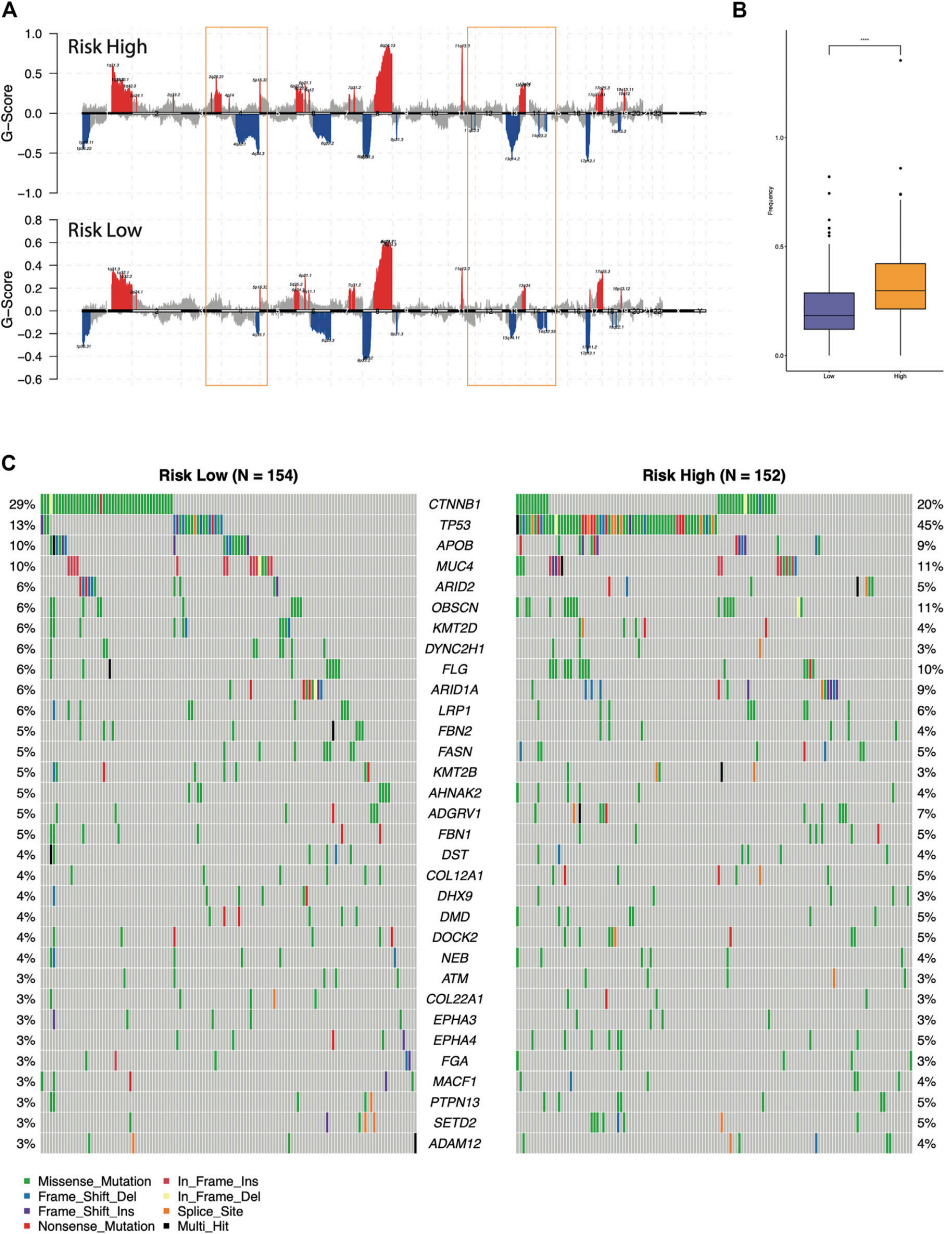
Comparison of the genomic alterations between the different risk groups. **(A)** Recurrent copy number aberrations of the high- and low-risk groups in the TCGA-LIHC cohort. Regions of amplifications (red) and deletions (blue) were above and below baseline (0.0), respectively. The orange box indicated the CNV regions with significant differences. **(B)** Comparison of the CNV frequency. **(C)** Oncoplot of the differential mutated AFs genes between high-risk and low-risk groups.

Furthermore, we compared the genomic aberrations between the different risk groups in TCGA-LIHC cohorts. The difference of copy number variation (CNV) was analyzed through maftools and GISTIC 2.0. As shown in Figures 5A,B, the high-risk group had significantly more deletion events and higher CNV frequencies than the low-risk group. Then, the differentially mutated AFs genes between the high-risk and low-risk groups were detected (Figure 5C, chi-squared test, *p*-value <0.05). Among them, the mutation frequency of HCC driver gene TP53 was enriched in the high-risk group (45 versus 13%, Fisher’s exact test *p*-value = 6.60e-10), this observation suggested the classic role of TP53 in cell-cycle regulation and guarding genome stability might also contribute to the malignant progression of HCC (Gao et al., 2019). While the CTNNB1 mutation was higher in the low-risk group than the high-risk group (29 versus 20%), the *p*-value slightly failed to reach statistical significance (Fisher’s exact test *p*-value = 0.063). Previously, multi-omics integration analysis revealed three HCC subtypes, one of which exhibited few CTNNB1 mutations companied by poor prognosis (Cancer Genome Atlas Resea, 2017). This was consistent with our observation that relatively lower mutation frequency of CTNNB1 in the high-risk group. In addition, the previous analysis also found that microvascular invasion was significantly reduced in the subtype with increased CTNNB1 mutation. We expected the mechanism of somatic mutations on angiogenesis in hepatocellular carcinoma to be further explored and verified.

### Potential Clinical Application of AFs Risk Score

**FIGURE 6:**
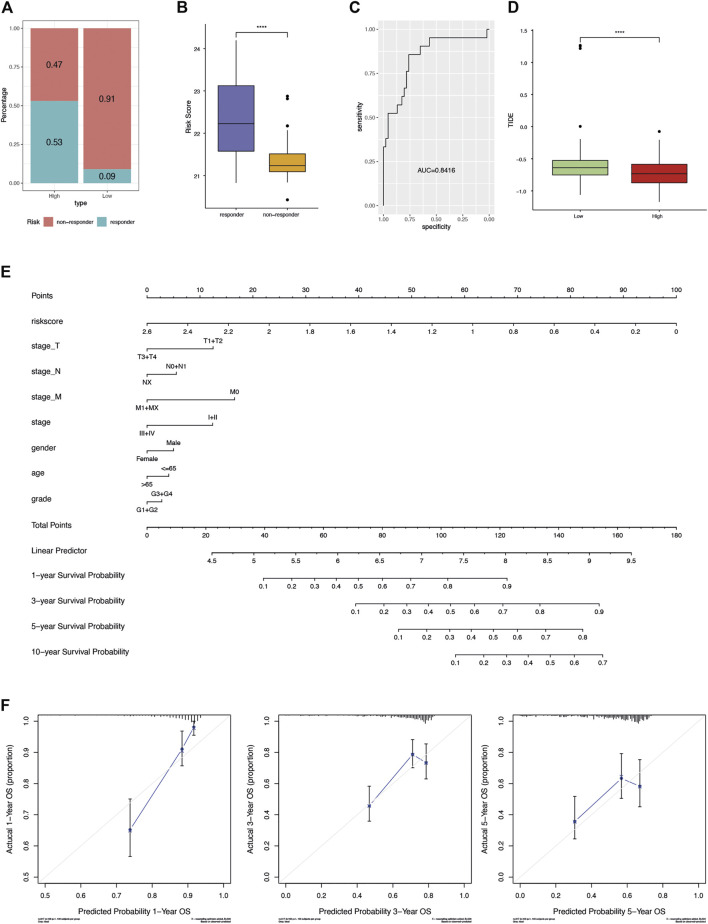
Potential clinical application of AFs risk score. **(A)** The proportion of samples responding to sorafenib in terms of the high- and low-risk groups. **(B)** The distribution of risk score between sorafenib responders and non-responders. **(C)** The ROC curve showed the sensitivity and specificity of risk score in predicting sorafenib response. **(D)** Boxplot compared the distribution of TIDE score between the high- and low-risk groups. **(E)** Nomogram-based AFs-derived prognostic model and clinical factors for 1-, 3-, 5- and 10-years OS prediction. **(F)** Calibration plot for agreement test between 1-, 3- and 5-years OS prediction and actual observation.

Accumulating evidence supported that sorafenib was effective in extending the time of progression in HCC (Vitale et al., 2010). In order to investigate the effect of our risk scoring model in HCC with sorafenib as adjuvant treatment, GSE109211 data set was used for analysis. The proportion of samples responding to sorafenib in the high-risk group was significantly higher than that in the low-risk group (0.53 versus 0.09, Figure 6A). Accordingly, the risk score was found extremely higher in responders (Wilcoxon rank-sum test *p*-value < 0.0001, Figure 6B). In other words, the risk score of the AFs prognosis model could effectively predict the patient’s response to sorafenib. The results of ROC curve analysis confirmed the good sensitivity and specificity of risk score (AUC = 0.8416, Figure 6C).

TIDE is the tumor immune dysfunction and rejection score, depicting the primary mechanisms of tumor immune evasion. It was proved to predict the clinical response and outcomes of patients following immunotherapy (Jiang et al., 2018). We used TIDE to evaluate the potential of risk score as a predictor of immunotherapy. Interestingly, the high-risk group showed significantly lower TIDE score (Wilcoxon rank-sum test *p*-value < 0.05, Figure 6D).

Finally, a graphic prognostic nomogram based on the 6-AFs genes was developed for 1-, 3-, 5- and 10-years prediction of OS for HCC patients from TCGA. The tumor stage, grade, age, and gender were also included (Figure 6E). Meanwhile, the calibration plot showed that the prediction by the nomogram had good agreement with actual observation (Figure 6F).

## Discussion

In the present study, we established an important prognostic model based on 6 DE-AFs genes significantly related to the prognosis of HCC, and further verified it in two independent validation datasets. The patients in the high-risk group showed poor prognosis, which was consistent in the three cohorts. Through univariate and multivariate Cox regression analysis, the risk score had significant prognostic value and was an independent prognostic factor of HCC. The model suggested that high risk may cause the regulation of immune mechanism, and these 6 gene signatures in the model could be used as potential prognostic molecular markers of AFs in HCC.

Several prognostic staging systems have been built for liver cancers, such as the Japan Integrated Staging score (Kudo et al., 2003), the Cancer of the Liver Italian Program score (Llovet and Bruix, 2000), the Tokyo Score (Tateishi et al., 2005), and the Barcelona Clinic Liver Cancer staging system (Llovet et al., 1999). These scoring systems mainly defined by tumor characteristics based on systemic literature reviews (Farinati et al., 2016). Some studies revealed that these scoring systems still lack substantial power for accurately predicting the survival of patients with liver cancer after curative resection, which may due to the high heterogeneity of liver cancer and lacking of molecular characteristics (Kim et al., 2014). Furthermore, hypervascularity and marked vascular abnormalities played a major role in tumor growth and spread of HCC (Morse et al., 2019). Thus, we constructed a machine learning model to distinguish patients survival using Least absolute shrinkage and selection operator (LASSO) regression. LASSO regression has obvious advantages in analyzing gene expression data due to the exists of multicollinearrity variables (McEligot et al., 2020). By adding L1 penalty, LASSO regression could effectively identify the most relevant variables for the outcome and reduce the dimensionality of the independent variables, reducing the effect of multicollinearity (Dai et al., 2021; Jia et al., 2021). By selecting an appropriate lamada, LASSO regression model could reduce model complexity and improve model prediction accuracy, resulting a good predictive efficiency for other datasets (Dai et al., 2021).

In this study, 6 DE-AFs genes were identified and included in the final prognostic model. The expression of *GRM8*, *SPC25*, and *FAM72A* was negatively correlated with favorable outcomes and also observed in other cancer types such as lung cancer (Zhang et al., 2019; Chen et al., 2018) and breast cancer (Wang et al., 2019). Recent research reports that the transcriptional activation of Metabotropic glutamate receptor 8 (*GRM8*) was elucidated to promote the survival of squamous cell lung carcinoma (LUSC) tumor cell through inhibiting cAMP pathway and activating MAPK pathway and the transcription level of GRM8 was reversely correlated with the prognosis of LUSC cases (Zhang et al., 2019), which is similar to our results. The upregulation of *SPC25* increased the cancer stem cell properties of non-small cell lung adenocarcinoma cells and was negatively correlated with survival (Chen et al., 2018). *SPC25* is also associated with poor prognosis in breast cancer patients (Wang et al., 2019). Zhang B *et al*’s study (Zhang et al., 2020) showed that *SPC25* overexpression promoted tumor proliferation and was a prognostic factor for a low survival rate of HCC, which is consistent with our results. FAM72A protein is overexpressed in several cancers (Guo et al., 2008). In a recent study based on the mice model, Rogier M *et al* found that the reduced levels of UNG2 mediated by overexpression of *Fam72a* would shift the balance towards mutagenic DNA repair, rendering cells more prone to acquire mutations (Rogier et al., 2021). In our study, there was a significant positive correlation between dryness and HRD score and risk score, and the high-risk group had significantly more missing events and higher CNV frequency. Among the 32 common high-frequency mutation information between high- and low-risk groups, the proportion of *TP53* mutation in high-risk group was significantly higher than that in the low-risk group (Figure 5). This echoes the standpoint that *TP53* is included in genes with frequent mutations in HCC (Totoki et al., 2014; Schulze et al., 2015; Chaisaingmongkol et al., 2017; Khemlina et al., 2017). The present results suggested that the identified DE-AFs signature was closely related to a worse prognosis of HCC. Therefore, the DE-AFs signature might be an easily applicable tool directing clinical decision-making.

The tumor microenvironment (TME) is a complex ecosystem consisting of various types of cells and the extracellular matrix with obvious heterogeneity (Maman and Witz, 2018). Tumor cell survival, growth, migration, and even dormancy are influenced by the surrounding TME (Biffi and Tuveson, 2021). Indeed, tumor angiogenesis is not only mediated by tumor cells, but also by cancer-associated fibroblasts (CAFs) and immune cells in the tumor stroma (Nyberg et al., 2008; Watnick, 2012; Mongiat et al., 2016). Increasing evidence suggests that solid tumors can be divided into hot tumors and cold tumors. Hot tumors are immune-inflammatory types characterized by adaptive immune activation, while cold tumors are immune rejection types characterized by innate immunity and interstitial activation (Turley et al., 2015; Chen and Mellman, 2017; Binnewies et al., 2018; Lin, 2021). We analyzed the difference of immune cell infiltration between high-risk and low-risk groups based on five independent HCC cohorts. The infiltration level of Treg cells with immunosuppressive effect and resting dendritic cells was positively correlated with the risk score, while most adaptive immune cells were just the opposite (Figure 4K). Our study revealed that the high-risk group may be more inclined to cold tumors. We also found follicular helper T cells (Tfh) showed a positive correlation with risk score in TCGA-LIHC cohort, while it was negatively correlated with risk score in GSE10141 and GSE10186 cohorts. This opposite situation may be due to the dynamic balance of various biological processes in organisms, or the role of immunosuppressive cells in the mechanism of immune escape, and further research is required.

Sorafenib is an oral multikinase inhibitor, its action mechanism includes inhibition of both MAPK/ERK-mediated cell proliferation and angiogenesis driven by VEGF signalling (Wilhelm et al., 2008). Sorafenib has been the standard systemic therapy for advanced HCC for a decade (Bouattour et al., 2019; Pinyol et al., 2019). In this study, among patients receiving sorafenib adjuvant chemotherapy, the proportion of non-responders in the low-risk group reached 91%. The results indicated that patients with higher risk score could benefit more from sorafenib, and it is recommended that patients with higher risk score undergo sorafenib adjuvant chemotherapy.

Nomograms have been widely used as prognostic devices in oncology and medicine (Balachandran et al., 2015; Song et al., 2018). Constructing a nomogram can transform the prediction model into a single factor of patient status evaluation, which provides effective support for personalized medical treatment for each patient. The nomogram of this study combined risk score, stage, gender, age, and grade, produced a favorable prediction effect. Although the impact of tumor heterogeneity on individual prognosis is still difficult to evaluate, the risk score as a practicable tool makes the nomogram more reliable and provides reference for clinical decision-making.

In summary, we constructed and validated a novel risk model consisting of 6 prognostic-associated AFs genes. This risk model showed effective and independent prognostic power, thereby providing important insight into the survival prediction of HCC. To our knowledge, this is the first study to predict prognosis of HCC patients based on the expression levels of AFs. Therefore, our study provided novel insights into the relationship between the regulation of AFs and development of HCC. In addition, we also revealed the underlying molecular features involving tumor stemness, immune regulation and genomic alterations between high/low-risk groups in this model. We expected further verification and mechanism exploration by the accumulated datasets in the future.

## Data Availability

The original contributions presented in the study are included in the article/Supplementary Material, further inquiries can be directed to the corresponding author.

## Supplementary Material

Supplementary Figure S1 Multivariate Cox regression analysis of the 17 prognostic candidate AFs in TCGA -LIHC cohorts.

Supplementary Figure S2 The expression level and KM curves of 17 angiogenesis factors with prognostic efficacy. Boxplots represent the expression level of angiogenesis factors in tumor and normal samples. Patients was separated using the median expression of each gene.

Supplementary Figure S3 Criteria for gene selection by LASSO regression analysis. **(A)** The cross-validation results and coefficient profiles of LASSO regression. For the left panel, the dotted line on the left indicated the value of log(λ) when the error of model is minimized, resulting six variables. For the right panel, the dotted line was drawn at the value chosen by cross-validation. **(B)** Expression correlation between TMC7 and genes included by the prognostic model. Spearman’s correlation test was performed.

Supplementary Figure S4 Distribution of GRM8 expression. **(A)** Density of GRM8 expression level. **(B)** Significantly differential expression of GRM8 in high and low risk groups (***, *p* value < 0.0001, Wilcoxon rank sum test).
