# Supplementary figures and images for: A Novel Prognostic Model for Identifying the Risk of Hepatocellular Carcinoma Based on Angiogenesis Factors
Source: Front Genet. 2022 Mar 18;13:857215. doi: 10.3389/fgene.2022.857215 (PMC8971657; doi:10.3389/fgene.2022.857215)

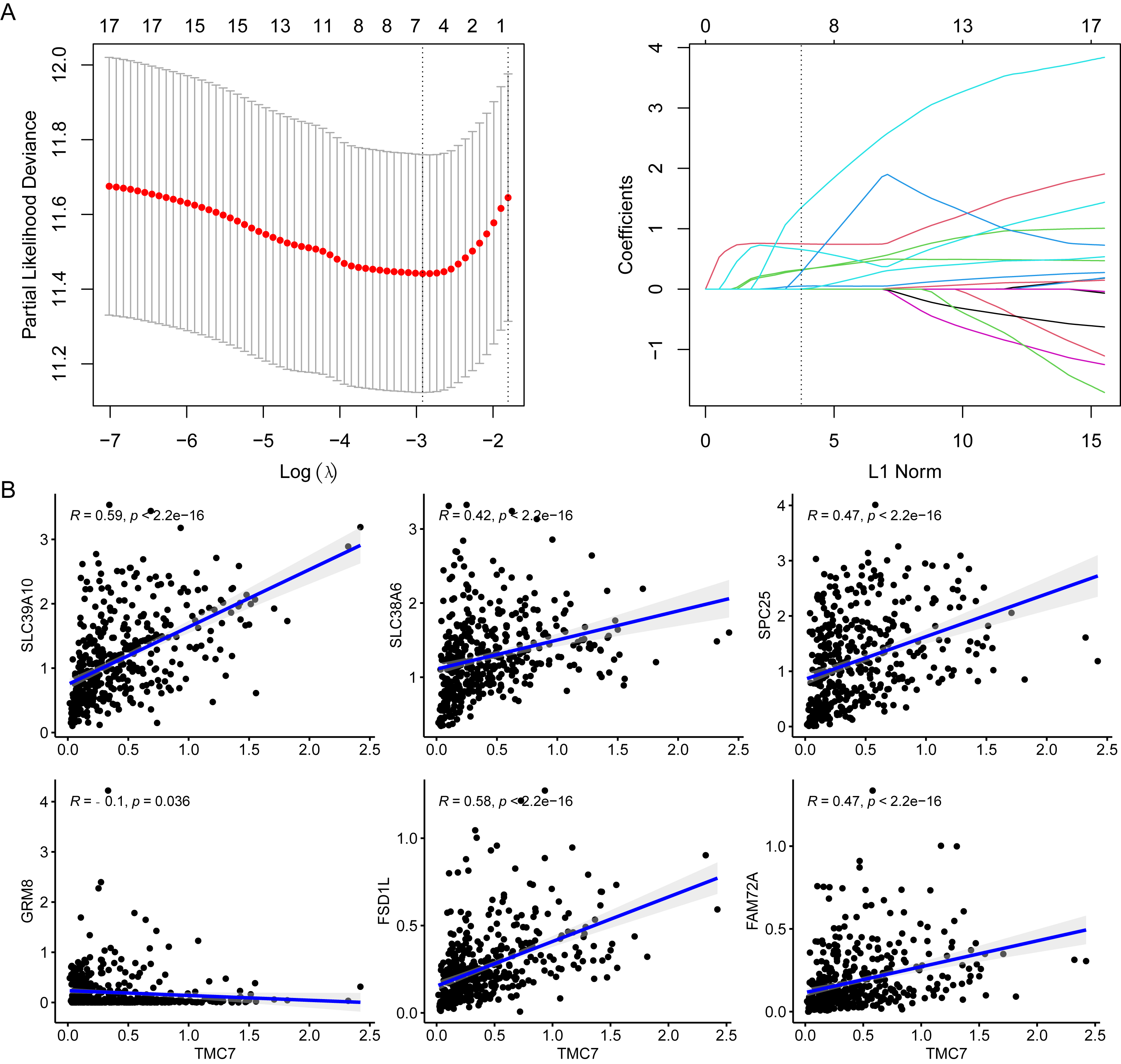

Supplement: Supplementary file 2 [file Image3.TIF]

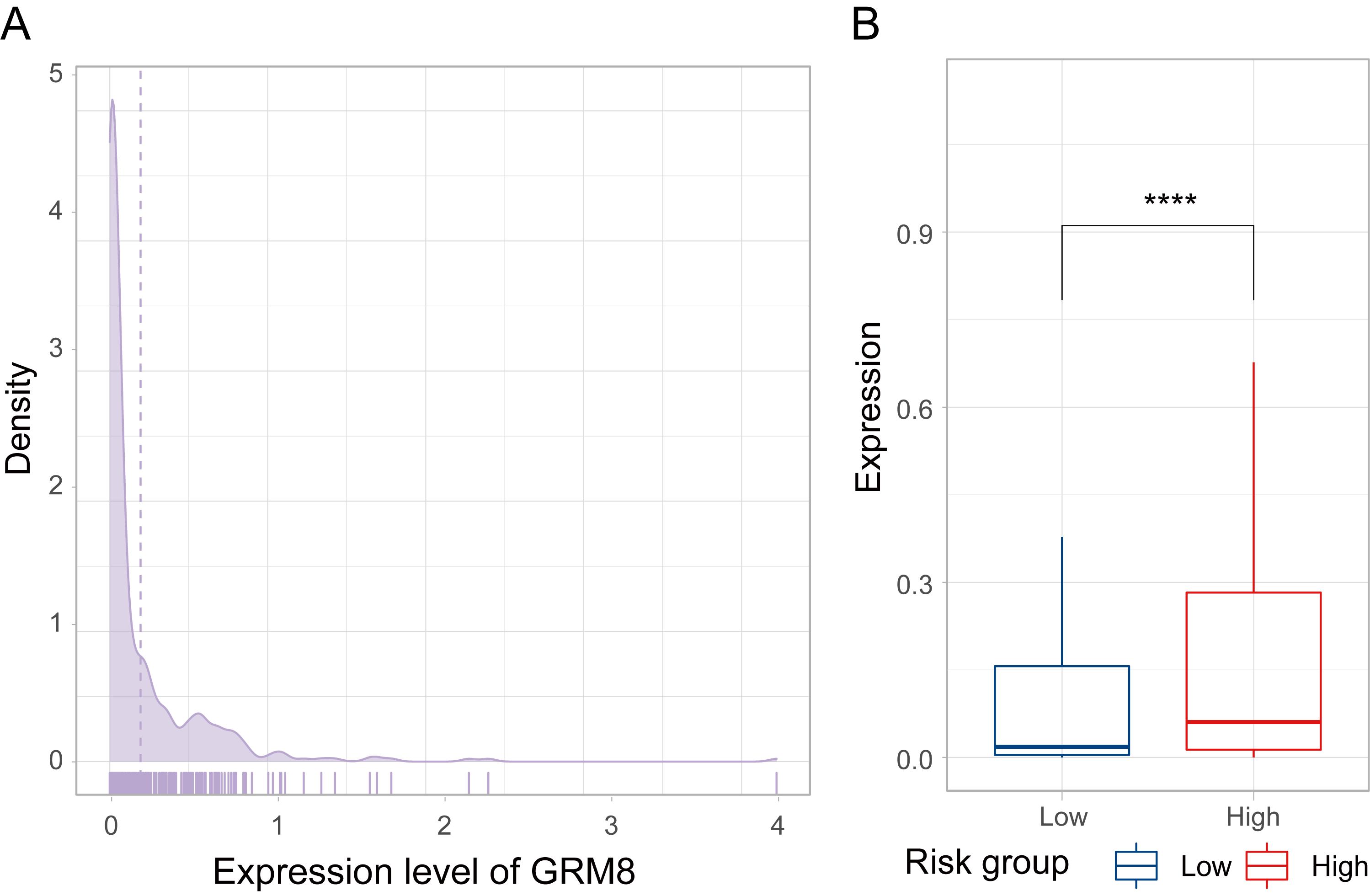

Supplement: Supplementary file 3 [file Image4.TIF]

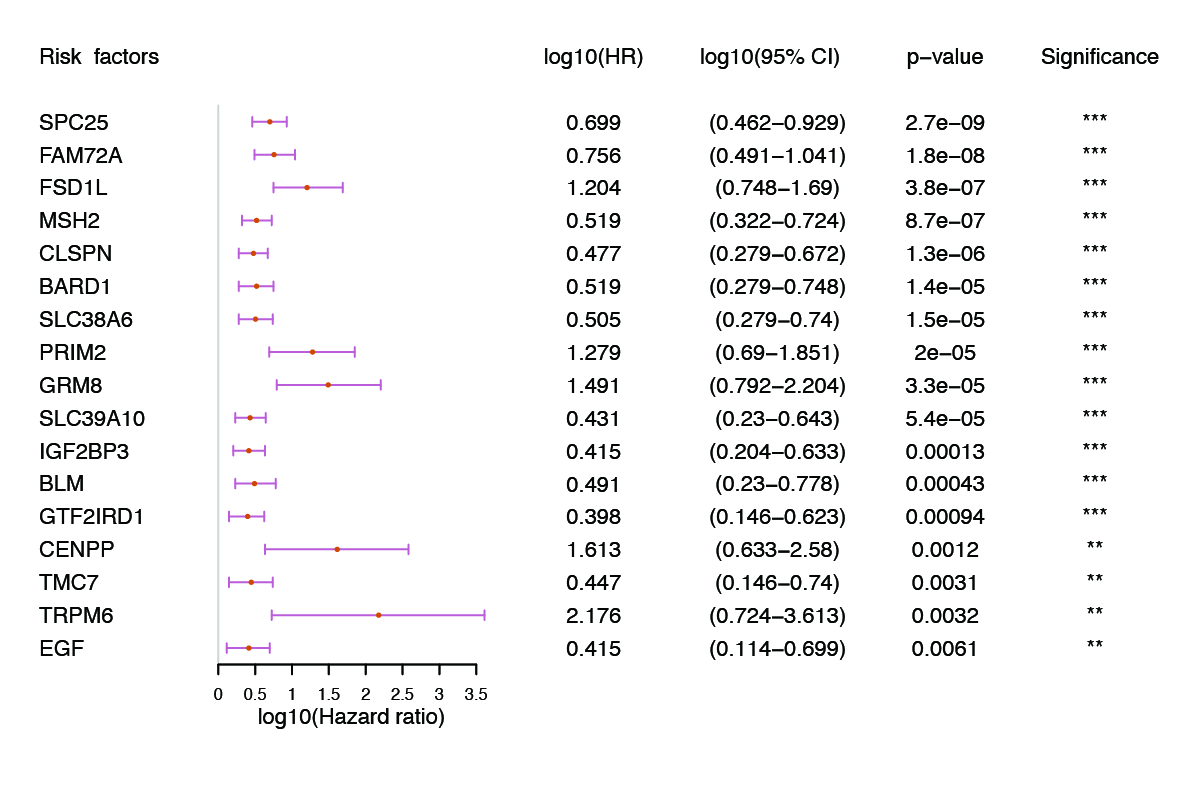

Supplement: Supplementary file 6 [file Image1.TIF]
